# Child and Adolescent Mental Health Service (CAMHS) Black Country Healthcare Foundation Trust (BCHFT) Trust-Wide Audit on Adherence to NICE Guidelines in Prescribing Medications for Children With Autism Spectrum Disorder (ASD)

**DOI:** 10.1192/bjo.2024.614

**Published:** 2024-08-01

**Authors:** Mazin Osman, Reka Ajay Sundhar, Helen Wheeldon, Zaynah Smith, Suma Ujrebail

**Affiliations:** Black Country Healthcare NHS Foundation Trust (BCHFT), West Midlands, Birmingham/Walsall, United Kingdom

## Abstract

**Aims:**

This comprehensive study seeks to evaluate the adherence of (CAMHS) service, Black Country Healthcare National Health Service (NHS) Foundation Trust to National Institute for Health and Care Excellence (NICE) guidelines in prescribing medications for children diagnosed with Autism Spectrum Disorder (ASD). Our primary objectives include identifying variations in prescribing practices across different localities within the trust and identifying specific areas that may benefit from improvement.

**Methods:**

A meticulous retrospective analysis was conducted on 142 randomly selected cases involving children diagnosed with ASD and prescribed psychotropic or sleep medications. A comprehensive analysis of patient records, encompassing progress notes and clinic letters, facilitated the gathering of extensive data. The evaluation centred around benchmarking adherence to NICE guidelines. Throughout the process, strict adherence to ethical standards was maintained.

**Results:**

Within the cohort of 142 children diagnosed with ASD, 44% underwent alternative interventions before medication initiation. Notable variations were observed across localities, with 87% receiving psychological therapy as an alternative intervention. Documentation of consent for commencing medication was present in 62% of cases. Specialists consistently initiated psychotropic medications at the minimum effective dose, and 70% of cases had a follow-up within 3–4 weeks. Sleep medications were prescribed to 77% of the cohort, with 55.5% of those undergoing alternative interventions before prescription.

**Conclusion:**

The study's findings underscore significant variations in adherence to NICE guidelines, emphasizing the critical importance of exploring alternative treatment modalities before resorting to medication. Furthermore, collaboration with supporting agencies is highlighted as a crucial aspect of comprehensive care. The documentation of consent forms for all patients is deemed imperative, and adherence to specified intervals for reviewing medication side effects, as outlined in the guidelines, is considered crucial for optimal and safe patient care.

